# Effect of invasive mechanical ventilation on the diversity of the pulmonary microbiota

**DOI:** 10.1186/s13054-022-04126-6

**Published:** 2022-08-22

**Authors:** Chang Liu, Kang Wu, Tianyu Sun, Bin Chen, Yaxing Yi, Ruotong Ren, Lixin Xie, Kun Xiao

**Affiliations:** 1 College of Pulmonary & Critical Care Medicine, 8th Medical Center, Chinese PLA General Hospital, Beijing, China; 2 School of Medicine, Nankai University, Tianjin, China; 3 MatriDx Biotechnology Co., Ltd, Hangzhou, China; 4 Foshan Branch, Institute of Biophysics, Chinese Academy of Sciences, Beijing, China

**Keywords:** Pulmonary microbiota, Invasive mechanical ventilation, Microaspiration, O_2_ toxicity, Ventilator-induced lung injury, Hyperoxia, Hypoxia, Single-cell RNA sequencing

## Abstract

Pulmonary microbial diversity may be influenced by biotic or abiotic conditions (e.g., disease, smoking, invasive mechanical ventilation (MV), etc.). Specially, invasive MV may trigger structural and physiological changes in both tissue and microbiota of lung, due to gastric and oral microaspiration, altered body posture, high O_2_ inhalation-induced O_2_ toxicity in hypoxemic patients, impaired airway clearance and ventilator-induced lung injury (VILI), which in turn reduce the diversity of the pulmonary microbiota and may ultimately lead to poor prognosis. Furthermore, changes in (local) O_2_ concentration can reduce the diversity of the pulmonary microbiota by affecting the local immune microenvironment of lung. In conclusion, systematic literature studies have found that invasive MV reduces pulmonary microbiota diversity, and future rational regulation of pulmonary microbiota diversity by existing or novel clinical tools (e.g., lung probiotics, drugs) may improve the prognosis of invasive MV treatment and lead to more effective treatment of lung diseases with precision.

**Supplementary Information:**

The online version contains supplementary material available at 10.1186/s13054-022-04126-6.

## Introduction

Healthy lungs serve as respiratory organs that accomplish O_2_/CO_2_ exchange between blood and external air. Due to less invasive sampling of healthy lungs and methodological limitations such as the ability to culture bacteria for complex biological samples [1], there has long been a lack of understanding of the microbiota of healthy lungs and even the assumption that healthy lungs (below the larynx) are sterile [2, 3]. In recent years, the rapid development and application of culture-independent molecular sequencing methods, especially high-throughput sequencing technologies (e.g., next-generation sequencing, NGS), have greatly facilitated the dynamic identification of microbial populations (microbiota) in various parts of the human body [4]. In 2010, Markus Hilty et al. did show the presence of bacteria in healthy lungs by 16S rRNA sequencing based on cytological brushes from the left upper lobe bronchoscopy of healthy lungs (approximately 2000 bacterial genomes/cm^2^ surface area), including bacterial genera such as Prevotella, Veillonella, Streptococcus and Haemophilus [5]. Follow-up studies further confirmed the presence of fungi (e.g., Cladosporium and Aspergillus spp.) [6] and viruses (e.g., anellovirus, Gardnerella phages and Lactobacillus phages) in healthy lungs as well by nucleic acid sequencing based on bronchoalveolar lavage fluids (BALFs) [7, 8]. The 10 most abundant genera (median proportion) in healthy lungs were Streptococcus spp., Prevotella spp., Veillonella spp., Haemophilus spp., Neisseria spp., Lacertus spp., Pseudomonas spp., Leptotrichia spp., Schaalia spp. and Candidatus Protochlamydia spp. (see Additional file 1: Table S1; 168 genera were detected in 1054 lung-associated samples at a median relative content of ≥ 0.01%) [9]. It is now generally accepted that the lung microbiota is established as early as the fetal period (the uterus is non-sterile) [10].

The pulmonary microbiota is vital for maintaining the functional balance of the lungs. It has been shown that the pulmonary microbiota is closely associated with the development and progression of different types of respiratory diseases, such as asthma [5, 11], chronic obstructive pulmonary disease (COPD) [12, 13], cystic fibrosis (CF) [14], non-CF bronchiectasis (BX) [15], tuberculosis [16, 17], novel coronavirus disease (COVID-19) [18, 19], lung cancer [20] and lung transplantation [6]. In addition, other external abiotic conditions such as smoking may also disturb the pulmonary microbiota [7].

The lung, as a semi-open organ, has a local microbial ecosystem of its own. The lungs are connected to the external environment through the airway, and along with each breath, the pulmonary microbiota also continuously exchanges microorganisms with the external environment and with the upper respiratory tract such as the oral cavity, nasal cavity, pharynx and trachea, thus creating a dynamic microbial cycle and a dynamic pulmonary microbiota. On the other hand, the pulmonary microbiota absorbs its required nutrients from the air phase (airways) and liquid/cellular phase (i.e., alveoli and their cellular components) of the semi-open biological niche (i.e., lungs) for dynamic self-renewal/maintenance. The lungs in whole, or their liquid/cellular phase confronting pulmonary microbiota directly, are armored with differential host defense arsenals functioning to affect pulmonary microbiota diversity [21]. Any biotic and/or abiotic stress that affects the biological niche and/or lung microbes may eventually interfere with the diversity of the pulmonary microbiota. Invasive mechanical ventilation (MV), a widely used method of life support in respiratory failure, may also be one such abiotic stress. In this review, we will systematically sort out the dynamics of pulmonary microbiota diversity in patients during invasive MV and then dissect the potential reasons why invasive MV affects pulmonary microbiota diversity.

## Invasive MV reduces the diversity of microbiota in lungs of adult patients

Often, the desire to study the effects of invasive MV on the human pulmonary microbiota requires the concurrent recruitment of non-invasive MV groups with comparable clinicopathophysiological characteristics. However, limited by issues such as clinical ethics, investigators often choose the more tractable method of longitudinal sampling and data analysis for the invasive MV group. Through longitudinal sampling and data observation of patients by timeline during invasive MV (Table 1), the currently available relevant clinical studies suggest that invasive MV may affect pulmonary microbiota diversity. In addition, most patients received concomitant antibiotic therapy before and/or during invasive MV, which may be a confounding factor in elucidating the effect of invasive MV on pulmonary microbiota diversity (Table 1).

**Table 1:** Clinical studies related to dynamic respiratory microbiota in patients with invasive MV based on NGS analysis of respiratory tract samples

Study design	Duration	Region (country)	Patients groups/numbers	Age (years)	Antibiotics	Samples from	Targets	Sequencing platform	Conclusion regarding to microbiota change during invasive MV	Study
NA	NA	America (USA)	Invasive MV patients (15)	≥ 18	Yes for all patients	Oropharynx and trachea	16S rRNA	MiSeq (Illumina)	Diminished bacterial diversity in the two kinds of samples from patients with invasive MV	Kelly et al. [22]
Post hoc analysis	Sep. 2008–Sep. 2010	Europe (mainly Spain)	Invasive MV with VAP (11); Invasive MV without VAP (18)	≥ 18	Yes for all patients	Trachea	16S rRNA	454 GS FLX + (Roche)	Diminished bacterial diversity in invasive MV patients	Zakharkina et al. [23]
Prospective	Dec. 2015–Nov. 2016	Europe (Switzerland)	Invasive MV with VAP (5); Invasive MV without VAP (5)	≥ 18	Yes for 8 patients	Oropharynx and trachea	16S rRNA	MiSeq (Illumina)	No significant change	Sommerstein et al. [24]
Prospective case control	Oct. 2012–Mar. 2014	Europe (Switzerland)	Invasive MV with VAP (18); Invasive MV without VAP (36)	≥ 18	Yes for virtually all patients	Oropharynx and trachea	16S rRNA	MiSeq (Illumina)	Diminished bacterial diversity in invasive MV patients	Emonet et al. [25]
Prospective	Jul. 2017–Aug. 2018	Asia (Korea)	Invasive MV with pneumonia (41); Invasive MV without pneumonia (19)	≥ 18	Yes for most patients	Trachea	16S rRNA	MiSeq (Illumina)	Diminished bacterial diversity in invasive MV patients with pneumonia; No relevant result reported in invasive MV patients without pneumonia	Woo et al. [26]
Prospective	Aug. 2014–Aug. 2018	America (USA)	Invasive MV with oral suctioning intervention (9); invasive MV without oral suctioning intervention (7)	≥ 18	Yes for most patients (14/16)	Oropharynx and trachea	16S rRNA	MiSeq (Illumina)	Diminished bacterial diversity in invasive MV patients	Sole et al. [27]
Prospective	As of Jul. 2020	America (USA)	Invasive MV COVID-19 with VAP (16); Invasive MV COVID-19 without VAP (17)	≥ 18	Yes for all patients	Trachea	Total RNA with human cytosolic and mitochondrial ribosomal RNA depleted	NovaSeq 6000 (Illumina)	Diminished bacterial diversity in invasive MV COVID-19 patients	Tsitsiklis et al. [28]
Post hoc analysis	November 2015-November 2016	Germany	30 patients in two groups were enrolled in the study: 15 patients with sepsis-induced ARDS following major abdominal surgery and 15 patients undergoing esophageal resection (serving as controls)	≥ 18	Yes for all patients	Bronchoalveolar lavage (BAL)	16S rRNA	MiSeq (Illumina)	Decreased alpha diversity was associated with increased duration of mechanical ventilation (= 0.48, *P* = 0.009) and diversity. Patients with ARDS had lower alpha diversity in BAL compared to controls (Shannon index 3 (2; 3.6) versus 1 (0.5; 1.5); *P* = 0.007)	Schmitt et al. 2020

NA: not available; *ARDS* acute respiratory distress syndrome

Here, we summarize the available clinical studies related to the dynamics of pulmonary microbiota diversity during invasive MV via NGS and show them in Table 1. In 2016, Kelly et al. found that the bacterial diversity of both samples collected within 24 h of invasive MV and samples collected after 24 h of invasive MV (invasive MV ≤ 14 days) was reduced [22]. In 2017, Zakharkina et al. compared the microbiota within tracheal aspirates of patients (≥ 18 years) at the onset of invasive MV and before extubation or at the development of VAP (≥ 7 days of invasive MV) based on 16S rRNA sequencing and found that during invasive MV there was indeed an antibiotic-independent reduction of bacterial diversity, and that this altered microbiota diversity was not associated with whether the patient developed ventilator-associated pneumonia (VAP) [23]. It is also noteworthy that patients with a final diagnosis of VAP had more variation in bacterial composition during invasive MV than did non-VAP patients [23]. In 2019, investigators longitudinally collected oropharyngeal swabs from adult patients (≥ four days of invasive MV) and analyzed patients' oropharynx during invasive MV by 16S rRNA sequencing, and no significant changes were found [24]. It is important to note that certain factors in this study, including the small number of patients recruited (five VAP patients and five non-VAP patients), the high sampling frequency (e.g., oropharyngeal swabs sampled daily), and the fact that the study was conducted on the oropharynx, i.e., the upper respiratory tract microbiota, rather than the lower respiratory tract (LRT) microbiota, may have contributed to the study not obtaining significant bacterial diversity changes [24]. In 2019, Emonet et al. found that bacterial diversity (by 16S rRNA sequencing) in oropharyngeal secretions (OPS) and endotracheal aspirates (ETA) decreased with increasing duration of invasive MV disposal in patients, regardless of the final diagnosis of VAP or non-VAP [25]. In 2020, Woo S et al. showed that for patients with severe pneumonia in the successful extubation and failed extubation groups, bacterial diversity was reduced in both their tracheal aspirates collected on day one and day seven of invasive MV when compared separately [26]. Furthermore, Sole et al. looked at the effect of oral suction intervention on changes in bacterial diversity in oral and tracheal samples from patients with invasive MV for more than five days and found that a reduction in bacterial diversity accompanying the time course of invasive MV (five time points, ≥ 12 h apart) was observed only in tracheal samples from patients with invasive MV without oral suction intervention, suggesting that the oral microbiota may contribute to the changes in the lower airway microbiota during MV [27]. In this study, seven patients experienced ventilator-associated conditions, one patient (control group) experienced an infection-related ventilator-associated condition, and cultures revealed Streptococcus pneumoniae and Staphylococcus aureus [27]. But all the comparison between MV patients with or without VAP has not been performed in this study. For COVID-19 patients, a reduction in diversity of the lower respiratory microbiota accompanying the time course of invasive MV (dividing 34 days of invasive MV duration into four consecutive time periods) was also found based on metagenomic analysis of tracheal aspirates during invasive MV, while a more significant reduction in microbiota diversity was observed in patients who eventually developed VAP [28].

To summarize the characteristics of the aforementioned studies (Table 1), it can be observed that (1) the patients recruited were all adults (≥ 18 years); (2) the patients were from the Americas (USA), Europe (Spain and Switzerland) or Asia (Korea); (3) most patients had antibiotic exposure during invasive MV; (4) biological samples were mainly from the oropharynx and/or trachea; (5) the vast majority of studies analyzed bacterial diversity by 16S rRNA sequencing; and (6) only one particular study analyzed the diversity of the overall microbiota (i.e., bacteria, fungi, and viruses) by metagenomics. The results of the vast majority of relevant studies showed that a progressive decrease in the microbial diversity of the patient's lower respiratory tract occurred during invasive MV (Table 1). In addition, the progressive decrease in microbial diversity in the lower respiratory tract of patients may be influenced or modulated by a variety of factors such as the duration of invasive MV and the microbiota of the upper respiratory tract such as the oral/oropharynx.

## Invasive MV reduces the diversity of microbiota in the lungs of neonatal or infant patients

The microbiome of full-term infants is similar to that of adults, consisting mainly of Streptococcus spp., Prevotella spp., Neisseria spp., Veillonella spp., Porphyromonas spp. and Fusarium spp. [29]. In contrast, the diversity of the respiratory microbiome of preterm infants is more susceptible to the mode of delivery due to their underdeveloped immune system and pulmonary physiology [29], so that the vast majority of neonates born by cesarean section at less than 35 weeks gestational age have a microbiota dominated by Staphylococcus spp., while those delivered vaginally have a microbiota dominated by Ureaplasma spp. [30]. The results of a prospective study with neonates showed that the microbial diversity of the lower airway was significantly lower during the first three days of mechanical ventilation, with a greater proportion of Klebsiella spp., Acinetobacter spp. and Streptococcus spp. composition that might indicate VAP [31]. Also, the investigators found that the microbiota composition in the endotracheal tube biofilm was more complex in neonates undergoing mechanical ventilation, and in particular that the abundance of Streptococcus spp. in the endotracheal tube biofilm was significantly associated with the development of VAP, the possible mechanism being that Streptococcus enhanced biofilm formation of Pseudomonas aeruginosa PAO1 and reduced IL-8 expression in airway epithelial cells exposed to PAO1 biofilm [32]. In addition, coagulase-negative staphylococci, gram-negative bacilli, methicillin-sensitive Staphylococcus aureus, Streptococcus viridans and Clostridium perfringens were more likely to be detected in preterm infants on long-term mechanical ventilation compared to those on short-term mechanical ventilation [33].

In another prospective study of children aged under 3 years admitted to a paediatric intensive care unit (PICU), Proteus spp. were the most abundant group of organisms in relative terms throughout the period of mechanical ventilation in paediatric patients, followed by Firmicutes spp. and Actinobacter spp. [34]. The alpha-diversity of the lower respiratory flora decreased along with increasing ventilator-associated infection (VAI) scores and days of mechanical ventilation. At the same time, the bacteria significantly associated with high VAI scores were oropharyngeal bacteria (they are not usually considered as paediatric VAI pathogens) [34]. This suggests that the ectopic migration of oropharyngeal bacteria may be related to the pathogenic mechanism of VAI. Furthermore, during mechanical ventilation for lower respiratory tract infections (LRTI) in children, the respiratory microbiota shifts from being dominated by Haemophilus spp. and Moraxella spp. to being dominated by antibiotic-resistant Enterobacteriaceae, Staphylococcus spp. and Streptococcus spp. [35].

## Concomitant events during invasive MV may drive progressive changes in pulmonary microbiota diversity at different levels

**Fig. 1:**
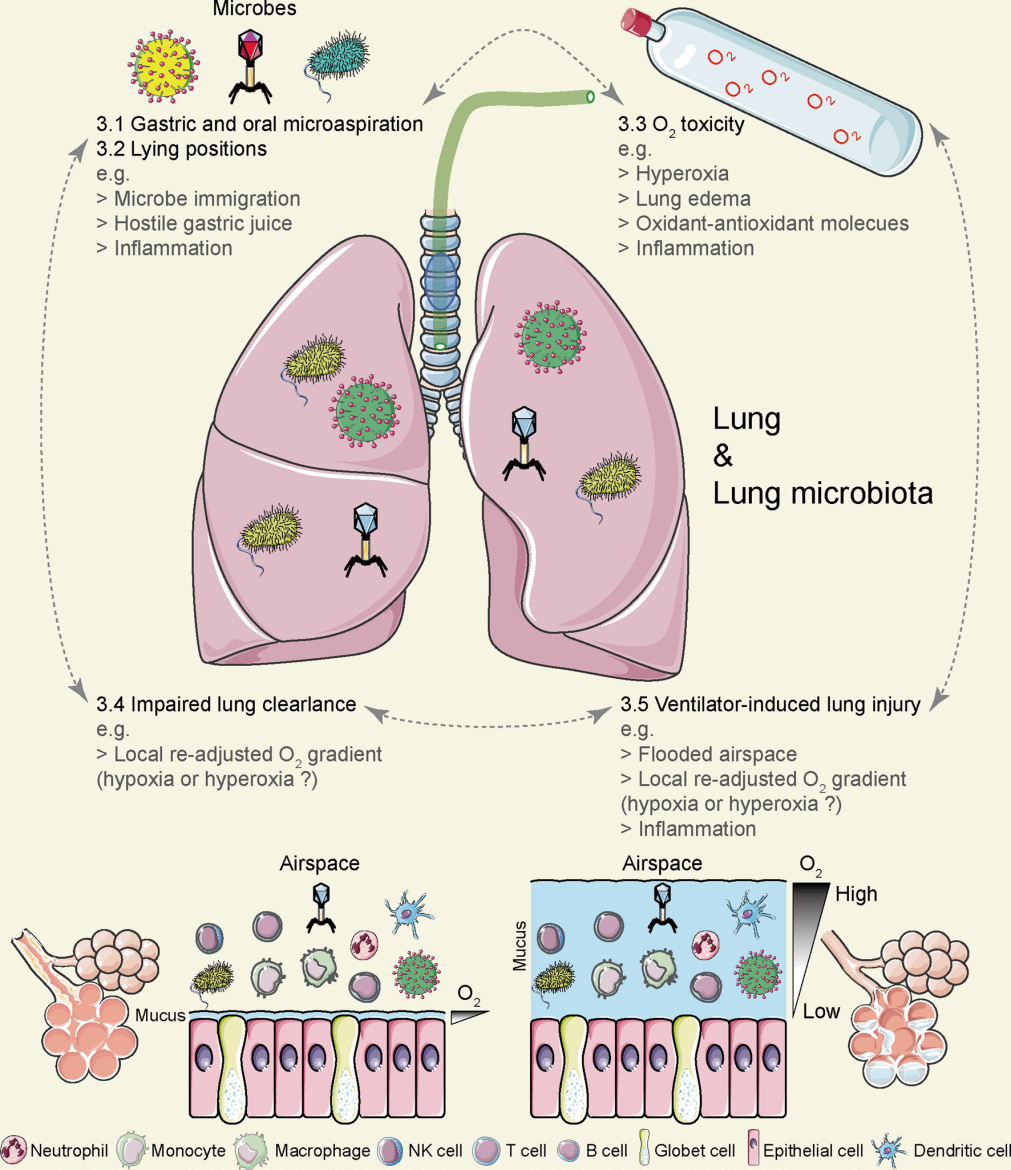
Concomitant events of invasive mechanical ventilation (MV) may affect pulmonary microbiota diversity through different mechanisms. Clinical disposition of patients using invasive MV is usually associated with a series of concomitant events, mainly including (1) gastric and oral microaspirations; (2) changes in body posture (prolonged recumbency); (3) oxygen toxicity due to high oxygen inhalation in hypoxemic patients; (4) impaired airway clearance; and (5) ventilator-induced lung injury (VILI). The above-mentioned invasive MV concomitant events may collectively affect the diversity of the pulmonary microbiota in a direct and/or indirect manner

Clinical disposition of patients using invasive MV is usually associated with a series of concomitant events, including (1) gastric and oral microaspirations; (2) changes in body posture (prolonged recumbency); (3) hemodynamic effects due to positive pressure ventilation; (4) complications of sedation, with or without paralysis; (5) O_2_ toxicity due to high O_2_ inhalation in hypoxemic patients; (6) disuse atrophy of the diaphragm; (7) impaired airway clearance; (8) ventilator-induced lung injury (VILI); and (9) other long-term effects (e.g., physical and mental health) after invasive MV disposition [36]. It is plausible to assume that patients who are neonates, children or adults could all experience these concomitant events. The above-mentioned invasive MV concomitant events may collectively affect the diversity of the pulmonary microbiota in a direct and/or indirect manner, and relevant (clinical) studies are sorted and described below (Fig. 1).

### Gastric and oral microaspirations during invasive MV might impact the diversity of pulmonary microbiota

Patients performing clinical management of invasive MV (both adults and infants) commonly encounter microaspiration of oropharyngeal secretions and/or gastric fluids [37]. In a two-year prospective multicenter study, the incidence of microaspiration measured by pepsin (a biomarker of gastric fluid microaspiration) in tracheal secretions of patients with invasive MV who received tube feeding could be as high as 88·9% (320/360) [38]. In another clinical study, by obtaining pepsin and amylase (biomarkers of oral microaspiration) in tracheal secretions, gastric microaspiration was found in seven of thirteen (54%) adult invasive MV patients receiving tube feeding and oral microaspiration in five patients (38%) [39]. In addition, it was also found that all 34 invasive MV preterm infants receiving tube feeding had gastric microaspiration by detecting pepsin levels in their tracheal aspirate [40]. It is evident that since different body sites/organ have their specific microbial signature components (i.e., characteristic microbiota) [9], it would be very common for patients to experience gastric and oral microaspiration during invasive MV, and this process would cause transfer of gastric microbiota and/or oral microbiota to the lungs [41, 42], which may ultimately affect the diversity of the pulmonary microbiota. Several researchers have done gastric fluid microaspiration with a rat model, resulting in a shift in the bacterial profile of the lungs from a predominance of Serratia spp., Ralstonia spp. and Brucella spp. to a predominance of Romboutsia spp. and Turicibacter spp. [43].

On the other hand, gastric microaspiration may affect pulmonary microbiota diversity in an indirect manner by exerting pathophysiological/inflammatory effects on the lungs. Numerous clinical studies and animal experimental evidence support the possibility that the acidity of gastric juice, the digestive enzymes (e.g., pepsin and bile salts) and pro-inflammatory molecules in the gastric juice may mediate a possible correlation between gastric microaspiration and acute/chronic airway disease as well as airway pro-inflammation [44, 45]. Based on a rabbit microaspiration model, investigators found that gastric fluid from invasive MV animals began to take a toll on the lungs of ventilated rabbits after six hours of microaspiration, as evidenced by a sharp and sustained decrease in PaO_2_/FiO_2_ and elevated lung inflammation (e.g., elevated neutrophil counts) [44]. Also, more inflammation-related molecules (e.g., IL-1α, IL-1β, and IL-8) were detected in gastric fluid samples from invasive MV animals at higher concentrations compared to surgically anesthetized controls [44]. Gastric juices from both control and invasive MV groups exhibited significant cytotoxicity in vitro, but showed different in vitro stimulatory abilities against human lung-derived epithelioid A549 cell line. A549 cells treated with gastric juice supernatant (pH approximately 7·45) from the invasive MV group were able to express more intercellular adhesion molecule-1 (ICAM-1) and IL-8 [44]. Different combinations of lung inflammatory cytokines may in turn cause changes in the diversity of the pulmonary microbiota. For example, pulmonary bacterial diversity was lower in BALF of children with *Mycoplasma pneumoniae* pneumonia compared to children with adenovirus pneumonia or tracheitis and was significantly associated with the expression of several inflammatory cytokines (i.e., IL-2, IL-4, IL-5, IL-6, TNF-α, and IL-1α) [46]. Briefly, the increased infiltration of inflammatory cells (e.g., neutrophils) caused an enhanced cross-talk between the lung cells and the microorganisms therein [47], while the increased inflammatory cytokines brought about a different remodeling of the ability of the lung cells to handle the microorganisms therein [48]. Moreover, the pathophysiological/inflammatory damage to the lungs by gastric juice may affect the dynamic growth of microorganisms in the lungs and thus the diversity of the pulmonary microbiota.

In conclusion, microaspiration during invasive MV may affect pulmonary microbiota diversity by immigrating the gastric/oral microbiota and/or causing lung damage in terms of pathophysiology/inflammation.

### Body recumbency during invasive MV may result in varying degrees of gastric microaspiration, leading to varying degrees of altered pulmonary microbiota diversity

By measuring the radioactive signal of endobronchial secretions (gastric fluid pre-labeled with technetium-99 m sulfur colloid) in 19 patients with invasive MV receiving tube feeding, Torres et al. found a continuous decrease in the signal detected when the patients were in the semi-recumbent position (45° angle) compared to the supine position (lying flat on the bed) [49]. In another study including 34 preterm infants with invasive MV who received tube feedings (half in the supine position; the other half first in the supine position; and then in the right side), by detecting pepsin levels in tracheal aspirates, lower gastric microaspirations were found in preterm infants in the supine plus lateral position compared to those in the supine position only [40]. The (semi-) lateral position can largely (or even completely) prevent gastric aspiration, which was verified in a vomiting-regurgitation situation using a commercial airway trainer manikin [50]. In conclusion, body recumbency during invasive MV can lead to varying degrees of gastric microaspiration, which in turn can cause alterations in the diversity of the pulmonary microbiota.

### O_2_ toxicity due to high levels of inhaled concentrations of O_2_ may lead to altered microbiota diversity in the lungs of patients with hypoxemia

Invasive MV with hyperoxia is a necessary clinical treatment for patients with (acute) hypoxemia, but it can also be accompanied by effects on the lungs and the microbiota therein. In fact, both short-term and long-term hyperoxia treatments can cause damage to the lungs. Studies based on rat models have shown that short duration (4 h) hyperoxia (FiO_2_ = 90%) induces pulmonary (oxidative) injury as evidenced by elevated lung pathology, increased pulmonary edema and disturbances in oxidant–antioxidant enzymes (i.e., glutathione reductase and xanthine oxidase) in BALF and lung tissue homogenates compared to conventional invasive MV [51]. Earlier studies suggested that compared to control rats (normoxia), adult rats exhibit a variety of abnormal injury-related phenotypes in their lung cells after prolonged exposure (14 days) to 85% O_2_, including time-related proliferation and hypertrophy of alveolar type II epithelial cells, death of a large number of capillary endothelial cells (41%) and a hypertrophic phenotype in surviving capillary endothelial cells after 7 days of hyperoxia treatment [52]. Meanwhile, the investigators obtained similar results in a mouse model. Mice exposed to hyperoxia (100% O_2_) also showed significant lung injury accompanied by upregulation of inflammatory cytokines (i.e., IL-6 and TNF-α), infiltration of macrophages and neutrophils, reduced activity of antioxidant enzymes (i.e., superoxide dismutase) and decreased reduced glutathione/oxidized glutathione ratio compared to mice exposed to normoxia [53]. In-depth clinical studies have shown that the lungs of healthy adults also show detectable changes after approximately 16·7 h of hyperoxia (> 95% O_2_) exposure through a padded mask, mainly in the form of increased plasma albumin and transferrin in BALF and activation of alveolar macrophages [54]. Furthermore, in vitro cytological studies in mice and humans and in vivo findings in mice suggest that increased extracellular vesicles (i.e., exosomes and microvesicles) in lung epithelial cells in response to hyperoxia promote pulmonary infiltration of macrophages and neutrophils, as well as inflammatory activation of macrophages [55, 56]. Based on the different states of inflammatory activation mediated by different cell types, extracellular vesicles and infiltrating inflammatory cells in the lungs in different states of activation (e.g., macrophages, neutrophils) are deeply involved in anti- or pro-microbial processes [57–59]. Therefore, it is conceivable to hypothesize that hyperoxia-induced pulmonary cellular responses may have an substantial impact on the diversity of the pulmonary microbiota.

On the other hand, the healthy lung microbiota contains both aerobic and anaerobic bacteria, and the composition of the lung microbiota of patients on the first day of mechanical ventilation is close to that of a healthy person. Half of the commensal flora of healthy lungs is composed of non-pathogenic anaerobic bacteria, 73% of which are strictly anaerobic, and these anaerobes are supposed to have a protective barrier role and play a key role in maintaining immune homeostasis in the lungs.[60–62]. The hyperoxic atmosphere (gas phase/gas environment) in the airway may stress the dynamic growth of the microbial complex of the lower respiratory (lung) microbiota. The relative abundance of bacteria in the lungs of mice exposed to hyperoxia (75% or 95%) changed significantly compared to normoxia, along with a significant decrease in bacterial diversity [63]. In vitro studies of different bacteria by Couvert et al. showed that the strict aerobic species of Pseudomonas fluorescens showed significantly slower growth when O_2_ concentrations were below 3% and showed a sharp growth arrest when O_2_ concentrations were below 1%. Two strict anaerobic strains of *Clostridium* (*C.*) *perfringens* and *C. sporogenes*, although still able to grow appropriately at lower O_2_ concentrations, stopped growth completely at O_2_ concentrations of 6·61% and 3·26%, respectively. In contrast, the other two parthenogenic anaerobic bacteria (*Listeria* (*L.*) *monocytogenes*) and *Bacillus* (*B.*) *weihenstephanensis* did not show growth changes in the range of O_2_ concentrations tested (0·1%–21% for *L. monocytogenes*; 0·1%–3·1% for *B. weihenstephanensis*) [64]. The results of Couvert et al. suggested that O_2_ concentrations below normoxia (21%) resulted in different bacterial strains, especially specialized aerobic/anaerobic species, exhibiting different degrees of growth variation. It can be inferred that hyperoxia during invasive MV may also trigger changes in microbiota diversity by altering the growth rate of complex microorganisms in the pulmonary microbiota, which warrants further relevant in vitro and clinical studies.

### Impaired voluntary airway clearance in patients due to invasive MV may affect the diversity of the pulmonary microbiota

Both cough clearance and mucosal ciliary clearance (MCC) contribute to airway clearance [65, 66]. However, airway clearance is often disturbed in critically ill patients. Any organic disorder that affects sternal structures, respiratory muscle function and its involved neurological functions (e.g., neuromuscular disease and spinal cord injury) may interfere with the effectiveness of coughing and thus reduce cough clearance [66]. Similarly, any disorder that affects the cellular components and overall function of mucosal ciliary (e.g., primary ciliary dyskinesia) can also eventually interfere with the MCC [65]. Closure of the glottis improves cough clearance to a great extent [67], but invasive MV keeps the vocal hilum open. As well, inappropriate humidification of inhaled gases, hyperoxia and trauma during aspiration that occurs with invasive MV can impair MCC function [68]. At the same time, routine care of patients with invasive MV (e.g., direct suctioning via an endotracheal tube) does not effectively clear the airway, especially the peripheral airways, resulting in the retention of pulmonary secretions [68] that are both endogenous (i.e., secreted by the respiratory unit below the tracheal cuff of the endotracheal tube) and exogenous via microaspiration of oropharyngeal secretions and/or gastric juices [37].

Secretion retention caused by impaired airway clearance may affect the diversity of the pulmonary microbiota in three ways.

1. Since exogenous secretions themselves contain their own microbiota, ectopic and retained exogenous secretions may affect the diversity of the local pulmonary microbiota through the exogenous microbiota they introduce.
2. For the local microbiota of the lower respiratory tract, retained endogenous or exogenous secretions may act as additional mediators/nutrients (beneficial or harmful), thereby inducing the differential growth of some microorganisms and ultimately leading to altered diversity in the local lung microbiota. For example, retained exogenous gastric fluid may have an acidic pH below 3, potentially complicating an already fragile neutral pH profile or pH gradient [69–71]. Furthermore, Quinn et al. found that a well-defined chemical gradient existed in the lung and that oxygen concentration and pH strongly partitioned the microbial community in a diseased human lung [72]. When the lung microbiota of CF patients was cultured in vitro in the pH range of 5-8.5, the low pH enriched for fermentative anaerobes, while the high pH enriched for parthenogenic anaerobes and microorganisms commonly considered pathogenic [72].
3. Trapped endogenous or exogenous secretions also additionally increase the thickness of the airway surface or its mucus layer, allowing for a wider gradient of O_2_ concentration regulation from the gaseous environment to the lining of the lung wall. The new O_2_ gradient (e.g., local hyper- or hypoxia) is undoubtedly a new stress on microorganisms and (infiltrating) inflammatory cells (e.g., alveolar macrophages and neutrophils) in the internal environment, on lung cells in the lung wall lining, and even on the cross-talk between microorganisms and lung cells. In the case of pathogenic *Mycobacterium tuberculosis* (*Mtb*) and human monocyte-derived macrophages (hMDMs), for example, although the extracellular viability of *Mtb* is comparable under normoxic and hypoxic conditions (1%), the growth rate of intracellular *Mtb* is significantly slower under hypoxic conditions (1%) compared to normoxia (partly, of course, due to hypoxia-induced upregulation of the antimicrobial peptide human β-defensin-2 expression) [73]. When exposing the lung microbiota of CF patients to a gradient of in vitro O2 saturation, Quinn et al. also found few microorganisms in the O2 saturated state and a more diverse anaerobic microbiota in the less saturated state [72].

### Ventilator-induced lung injury (VILI) could impact pulmonary microbiota diversity

VILI arises from a variety of mechanical traumas during invasive MV, including volutrauma, barotrauma and atelectrauma [74]. Volutrauma is caused by alveolar hyperinflation and manifests mainly as fluid-filled air spaces containing activated macrophages and neutrophils due to increased permeability of the alveolar–capillary barrier (pulmonary edema). Barotrauma is caused by higher trans-pulmonary pressures (i.e., alveolar pressure values minus intrathoracic pressure) and manifests mainly as pneumothorax, pneumoperitoneum and complications due to alveolar rupture (air leak) resulting in complications such as subcutaneous emphysema. Atelectrauma is caused by higher shear forces and is mainly manifested by noxious shear stress and strain on the epithelium (airway epithelium) at the interface between the air bubble and the airway due to periodic hemorrhagic opening/collapse of the recruitable lung units. The above mechanical trauma or subclinical mechanical trauma is often accompanied by the generation of an associated biological response characterized by upregulation of expression of pro-inflammatory mediators/cytokines (e.g., TNF-α, IL-1β, IL-6, IL-8), infiltration of leukocytes (e.g., activated macrophages and neutrophils), and perhaps inflammation-mediated pathological injury. Therefore, this (subclinical) trauma-derived series of biological events is also defined as biotrauma.

We hypothesized that VILI may affect the diversity of the pulmonary microbiota through three aspects: (1) flooding of the airspace with fluid; (2) infiltration of blood leukocytes; and (3) damage to airway epithelial cells. As mentioned before, the submerged fluid consists of endogenous or exogenous secretions, which on the one hand can serve as an additional medium/nutrient (beneficial or harmful) for local microorganisms and on the other hand implies additional fluid thickness/depth (i.e., sets a wider O_2_ gradient for local microorganisms/airway endothelial cells/infiltrated leukocytes). At the same time, the infiltrating blood leukocytes (e.g., macrophages and neutrophils) create a complex cross-talk with local microorganisms and even free material such as cellular debris from airway epithelial cells, which in turn induces changes in the local immune microenvironment and ultimately affects the local microbiota diversity of the lung.

## Local immune responses in the lung due to O2 concentration perturbations may also somehow regulate changes in the diversity of the pulmonary microbiota

**Fig. 2:**
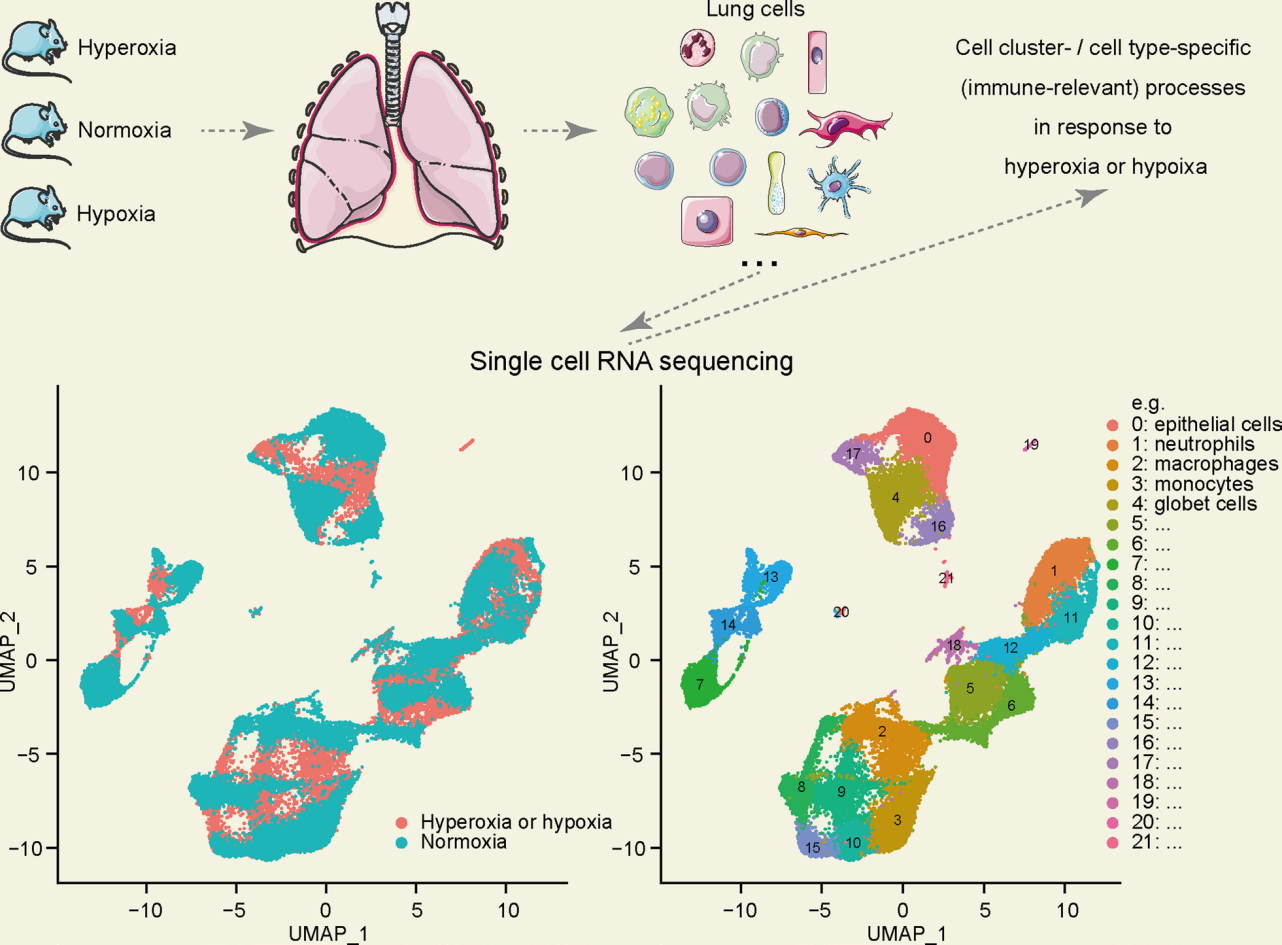
Mouse studies based on single-cell transcriptome analysis suggest that hyperoxia or hypoxia can modulate the local immune microenvironment of the lung. These findings suggest a dynamic interplay between microbiota and host immunity and give us reason to hypothesize that perturbations in local lung oxygen concentration originating from invasive MV may alter the diversity of the pulmonary microbiota by affecting the local immune microenvironment. UMAP: Uniform Manifold Approximation and Projection

As mentioned above, the patient's lungs may also experience local O_2_ concentration perturbations (hyper- or hypoxia) during invasive MV. Given that lung cells contain more than 40 different cell types, the effect of O_2_ concentration perturbations on the local immune microenvironment of the lung cannot be studied by high-throughput sequencing methods targeting mixed cell samples such as lung tissue, alveolar lavage fluid and airway aspirates [75]. A series of recent studies have revealed in depth by scRNA-seq techniques that lung cells generate local immune responses to hyperoxia or hypoxia, causing changes in the local immune microenvironment of the lung. A few of these studies identified different patterns of lung cell immune responses that can be triggered by hyperoxia [76, 77] and hypoxia [78–80], respectively (Table 2, Fig. 2). Hurskainen et al. performed single-cell transcriptome analysis of lung tissue from neonatal mice (P0) after three, seven or 14 days of exposure to normoxia or hyperoxia (85% O_2_) (i.e., P3, P7 or P14) and found that hyperoxia exposure specifically activated immune-related signaling pathways in several cell populations/cell types (Table 2) [76]. Among them, positive enrichment of signaling pathways included “activation of NF-kb in B cells” and “NIK NF-kB signaling” in type-2 alveolar epithelial cells (AT2); “inflammatory response,” “TNF-related signaling pathway” and “innate immune response” in stromal cells; “response to type I interferon” in endothelial cells response,” “humoral immune response” or “antigen processing cross-expression” for endothelial cells; “inflammatory response,” “regulation of leukocyte migration” for alveolar macrophages; “response to gamma interferon” and “antigen processing and presentation via MHC I” for interstitial macrophages; “positive regulation of cytokine secretion,” “positive regulation of leukocyte chemotaxis,” “cellular response to interleukin-1” for neutrophils, “regulation of chemokine production” and “antigen processing and presentation”; “cross-expression of antigen processing” for B cells; and “interferon gamma response” and “response to interferon gamma” for CD8 + T cells [76]. On the other hand, negative enrichment of signaling pathways includes “negative regulation of inflammatory response” in B cells and “leukocyte differentiation” in CD8 + T cells [76]. In another study, Wu et al. explored changes in lung cell-specific gene expression in intermittently hypoxic mice (eight–ten weeks of age) based on single-cell transcriptome sequencing technology (Table 2) [80]. Their findings revealed that positive enrichment of the “response to TNF/interleukin-related processes” signaling pathway was observed in lymphatic endothelial cells, myofibroblasts, basophils, macrophage-dendritic CD163 + cells, and dendritic cells in the lung. Negative enrichment of signaling pathways such as “immune response-related,” “antigen processing and expression,” “response to interferon,” “chemotaxis,” “phagocytosis” and “response to cytokines” can be observed in erythrocytes, basophils, macrophage-dendritic CD163 + cells, dendritic cells, macrophages, monocytes, T cells and natural killer T cells. These findings, combined with other existing studies suggesting a dynamic interplay between microbiota and host immunity, give us reason to hypothesize that perturbations in local lung O_2_ concentration originating from invasive MV may alter the diversity of the pulmonary microbiota by affecting the local immune microenvironment [81–83].

**Table 2:** Related studies on changes in the local immune microenvironment of the lung due to O_2_ concentration perturbation based on scRNA-seq technique, which in turn affects the diversity of the pulmonary microbiota

Species	Age	Gender	Study design	Samples	Sequencing platform	Transcriptionally distinct cell clusters	Immune-relevant events due to changed O_2_ concentration exclusively	Study
Mice (C57BL/6)	Postnatal day (P) 0	Random	Normoxia or hyperoxia (85% O2) for 3 days (P3), 7 days (P7), or 14 days (P14), and subsequent lung cell preparation for scRNA-seq; 6 mice per group, 36 in total	Whole lung	NextSeq500 (Illumina)	34 clusters	The study described cell cluster- /cell type-specific transcriptional analysis in response to hyperoxia, compared to normoxia (via gene set enrichment analysis (GSEA))	Hurskainen et al. [76]
Mice (C57BL/6 J)	Postnatal day (P) 0	Random	Normoxia or hyperoxia (> 95% O2) for 3 days, and lung cell preparation for scRNA-seq on P7 and P60	Whole lung	HiSeq (Illumina)	45 clusters	The study did not analyze the cell cluster-/cell type-specific transcriptional analysis in response to hyperoxia, compared to normoxia	Scaffa et al. [77]
Mice (C57BL/6 J)	8–10 weeks	Male	12 h:12 h light/dark cycle for 2 weeks, then intermittent hypoxia (6%, ~ 30 hypoxic events/h; *n* = 3) or normoxia (*n* = 3) for 9 days, then scRNA-seq next day	Whole lung	HiSeq 2500 (Illumina)	25 clusters	The study described cell cluster- /cell type-specific transcriptional analysis in response to hypoxia, compared to normoxia (via biological process enrichment analysis in the DAVID database)	Wu et al. [80]
Mice (CDH5-CreERT2-ROSA26-TdTomato, endothelial cell-specific Cdh5-driven expression of Td Tomato red fluorescent protein)	NA	Female	Gavaging tamoxifen for TdTomato induction. Then hypoxia (10% O2 for 3 weeks)/weekly subcutaneous injection of 20 mg/kg Sugen 5416 (*n* = 3), or normoxia for 3 weeks. Then sorting TdTomato + mouse lung cells for scRNA-seq	TdTomato + mouse lung cells (endothelial cells)	NovaSeq S2 (Illumina)	8 clusters	Due to the injection of Sugen 5416 in hypoxia group (not in normoxia group), so the study design could not provide hypoxia-exclusive effect on endothelial cells	Rodor et al. [79]
Rats (Sprague–Dawley)	Adult (250 g–350 g)	Male	Subcutaneous injection of 20 mg/kg Sugen 5416 followed with 10% O2 for 21 days and the normoxia for 14 days (*n* = 6); Normoxia control were kept for 28 days (*n* = 6)	Whole lung	HiSeq 4000 (Illumina)	28 clusters	Due to the injection of Sugen 5416 in hypoxia group (not in normoxia group), so the study design could not provide hypoxia-exclusive effect on lung cells	Hong et al. [78]

## Conclusion

As mentioned above, a series of basic and clinical studies have helped us to understand the impact of invasive MV and its associated concomitant events on the diversity of the pulmonary microbiota and its associated biotic (physiological, cellular and molecular) and abiotic (physical, chemical) mechanisms. These studies may even help us to understand at the single-cell transcriptomic level how the perturbation of O_2_ concentrations (i.e., hyper- or hypoxia) that accompanies invasive MV affects lung cell function, how it alters the local immune microenvironment of the lung and how it ultimately induces changes in the diversity of the local pulmonary microbiota. Of course, more in vitro and in vivo studies are needed to provide a clearer validation of the above biological processes and their occurrence mechanisms. For example, (1) we should establish stable and reliable in vitro culture/maintenance models of the pulmonary microbiota to enable a clearer understanding of the mechanisms by which variables such as gastric pH and O_2_ concentration gradients regulate pulmonary microbiota diversity. (2) We should acquire representative strains of the pulmonary microbiota, including aerobic and anaerobic microorganisms, so that we can potentially obtain a clearer understanding of both their interactions and their relationship with the pulmonary microbiota through a series of in vitro studies, and their interactions and their cross-communication with lung cells (e.g., pulmonary epithelial cells, alveolar macrophages, infiltrating neutrophils) and even the local lung microenvironment through a series of in vitro studies.

Once we have a deeper understanding of the relevant cellular and molecular mechanisms, new clinical approaches (e.g., pulmonary probiotics, drugs) can be complemented during the treatment of invasive MV, thus maintaining a benign balance of pulmonary microbiota diversity while further exploring how to regulate pulmonary microbiota diversity for a more scientific and efficient clinical management of the therapeutic process of pathogenic diseases [84]. Some conceptual therapeutic directions include (1) inhalable pulmonary probiotics to maintain a healthy lung microbiota, (2) inhalable drugs specifically/primarily targeting microinhalation of stomach- or mouth-specific microorganisms during invasive stroke, (3) inhalable inflammatory modulators to reduce O2 toxicity or VILI and (4) rational invasive MV procedures to reduce exogenous or endogenous fluid retention and thereby alleviating local O2 concentration perturbations. Currently, an encouraging finding suggests that viable bacteria (i.e., mixed human oral commensal Prevotella melaninogenica, Veillonella parvula and Streptococcus mitis) can be successfully inhaled into LRT of a mouse model, in modulating the lung immune phenotype and attenuating host susceptibility to Streptococcus pneumoniae [85]. Despite this, to date, no clinical studies have really clarified the feasibility of lower respiratory “respiratory probiotic” therapy (aerosolization of viable microbiota into the lower respiratory tract), and both its safety and potential adverse effects must be supported by convincing evidence before it can enter the clinical translation process. In addition, future research will need to focus on important issues such as microbial–host interactions and inter-microbial interactions, the safety of probiotic therapy in vulnerable and high-risk patients such as children, critically ill patients and immunodeficient patients and the optimal timing for modulating the microbiota.

## Supplementary Information

**Additional file 1:** Full list of 168 genera detected in ≥ 2 out of 1054 lung-relevant samples with median relative abundances ≥ 0.01%. (Accessed the website of ‘https://mbodymap.microbiome.cloud/#/mbodymap’  on July 3, 2022).

## Data Availability

Not applicable.
