# Supplementary material for: Effect of invasive mechanical ventilation on the diversity of the pulmonary microbiota
Source: Crit Care. 2022 Aug 22;26:252. doi: 10.1186/s13054-022-04126-6 (PMC9394019; doi:10.1186/s13054-022-04126-6)
Supplement: Supplementary file 1 — Additional file 1: Full list of 168 genera detected in ≥ 2 out of 1054 lung-relevant samples with median relative abundances ≥ 0.01%. (Accessed the website of ‘https://mbodymap.microbiome.cloud/#/mbodymap’ on July 3, 2022). [file 13054_2022_4126_MOESM1_ESM.pdf]

Name	ncbi_taxon_id	relative_abundance_avg (%)	relative_abundance_med (%)	relative_abundance_std	relative_abundance_sum
Streptococcus	1301	25.15195	20.24485	20.09812	26510.15039
Prevotella	838	15.6905	13.508	12.63123	16459.32984
Veillonella	29465	11.78943	10.6363	8.14652	12272.80128
Haemophilus	724	7.21314	3.71328	9.23792	7501.66126
Neisseria	482	8.43433	3.47866	10.85882	8788.57161
Lacibacter	649461	3.12483	3.12483	2.65985	6.24965
Fusobacterium	848	3.90686	2.56794	4.32871	4035.79091
Leptotrichia	32067	2.68297	1.24867	3.84343	2752.73029
Schaalia	2529408	1.85463	0.99085	2.29074	1902.84671
Candidatus Protochlamydia	282132	0.82377	0.82377	0.81263	1.64755
Bacteroides	816	1.52985	0.81841	2.45102	1552.79988
Gemella	1378	1.68118	0.68913	2.86652	1745.05994
Enterococcus	1350	1.06001	0.68888	1.17696	1047.29456
Chroococcidiopsis	54298	0.65772	0.65772	0.61457	1.31545
Porphyromonas	836	1.6916	0.6186	2.92082	1742.35142
Rothia	32207	1.53047	0.56631	2.64651	1573.32201
Actinomyces	1654	1.10177	0.56311	1.53581	1137.02951
Atopobium	1380	1.22406	0.4856	1.68415	1206.92322
Pseudoxanthomonas	83618	0.52272	0.39201	0.51433	7.84085
Planctopirus	1649480	0.34917	0.34917	0.33556	0.69834
Sulfurihydrogenibium	212790	0.298	0.298	0.28122	0.59599
Selenomonas	970	0.67822	0.28021	1.1636	678.21578
Nonlabens	363408	0.30272	0.23461	0.32797	2.72447
Acidiphilium	522	0.22521	0.22521	0.2062	0.45041
Allochromatium	85072	0.1989	0.1989	0.17078	0.3978
Lachnoanaerobaculum	1164882	0.44953	0.18886	0.66177	434.24229
Sphingobium	165695	0.7159	0.1733	1.73899	30.78356
Curvibacter	281915	0.29927	0.17264	0.32792	5.68613
Capnocytophaga	1016	0.66568	0.16071	1.68223	670.34005
Tepidiphilus	203470	4.25342	0.13974	8.37414	127.60265
Rickettsia	780	0.46421	0.13887	0.62546	11.60519
Gelidibacter	49279	0.13107	0.13107	0.11565	0.26215

Castellaniella	359336	0.12246	0.12246	0.12212	0.24491
Bulleidia	118747	0.2161	0.11152	0.33091	213.29377
Granulicatella	117563	0.29736	0.10729	0.577	306.58009
Borreliella	64895	0.52153	0.10136	0.72101	4.69373
Cryobacterium	69578	0.10028	0.10028	0.01804	0.20055
Tessaracoccus	72763	0.1838	0.09868	0.21917	0.73521
Campylobacter	194	0.24072	0.09756	0.37592	238.55764
Aggregatibacter	416916	0.32386	0.09035	0.81824	312.52673
Entomoplasma	46238	0.11026	0.08292	0.09689	1.32317
Vulcaniibacterium	1649512	0.34956	0.07571	0.73841	2.79649
Fischerella	1190	0.11958	0.07447	0.14929	1.67417
Nitratifractor	269260	0.1473	0.07099	0.16094	3.38784
Herbihabitans	1884922	0.06902	0.06902	0.00195	0.13804
Candidatus Midichloria	411566	0.1155	0.06522	0.11728	0.57749
Salinimicrobium	561367	0.06337	0.06337	0.06259	0.12674
Niabella	379899	0.32891	0.06167	0.42234	2.30239
Peptostreptococcus	1257	0.20812	0.05851	1.17143	191.88809
Thermomonas	141948	0.46384	0.05642	0.76017	7.42145
Massilia	149698	0.06443	0.05619	0.05282	0.38658
Alloprevotella	1283313	0.33268	0.05555	0.74819	293.42506
Collimonas	202907	0.07805	0.05514	0.05631	0.54635
Weeksella	1013	0.05464	0.05464	0.04167	0.10929
Gloeocapsa	102231	0.07338	0.05258	0.07804	0.66043
Microvirga	186650	0.06527	0.05251	0.05612	0.26107
Turneriella	338321	0.04743	0.04743	0.0471	0.09486
Acidovorax	12916	3.28823	0.04674	8.92485	220.31118
Variovorax	34072	0.40485	0.04612	0.96248	41.295
Sandarakinorhabdus	362865	0.04527	0.04527	0.01775	0.09053
Borrelia	138	0.04372	0.04372	0.02727	0.08744
Parvimonas	543311	0.17004	0.04127	0.52215	157.11648
Nubsella	565005	0.04069	0.04069	0.03987	0.08138
Phaeobacter	302485	0.04021	0.04021	0.0087	0.08042
Treponema	157	0.24217	0.03891	0.80258	218.19538
Thermoanaerobacterium	28895	0.5428	0.03875	1.06779	5.42805

Aeribacillus	1055323	0.17911	0.03853	0.34231	3.94043
Methanothermococcus	155862	0.03548	0.03548	0.03468	0.07097
Brucella	234	0.4971	0.03537	1.39728	96.93387
Catonella	43996	0.07817	0.03455	0.2063	72.93028
Leptospirillum	179	0.03435	0.03435	0.03344	0.06869
Propionimicrobium	203133	0.04944	0.03389	0.04844	0.24718
Methanococcus	2184	0.03332	0.03332	0.0144	0.06663
Sulfuricurvum	286130	0.15968	0.03309	0.21339	2.3952
Corynebacterium	1716	0.247	0.03237	1.56753	240.57743
Zhizhongheella	1649514	0.12682	0.03213	0.18047	0.63411
Chishuiella	1572663	0.03206	0.03206	0.02418	0.06411
Bdellovibrio	958	0.15238	0.03171	0.34682	2.74284
Halopelagius	1008195	0.03161	0.03161	0.01155	0.06321
Dialister	39948	0.09807	0.0313	0.19997	90.80977
Thermobifida	83677	0.11798	0.03043	0.14776	4.01116
Thermithiobacillus	119979	0.02961	0.02961	0.01969	0.05921
Flectobacillus	101	0.60552	0.02786	1.47119	11.50482
Halomonas	2745	0.3061	0.02764	1.15497	16.52923
Runella	105	0.02721	0.02721	0.02679	0.05443
Mitsuaria	65047	0.02555	0.0267	0.01686	0.2044
Ohtaekwangia	1210119	0.05343	0.02629	0.06355	0.37402
Butyrivibrio	830	0.11429	0.02588	0.23029	82.63167
Novosphingobium	165696	0.54392	0.02563	1.35723	31.00337
Agrobacterium	357	0.08284	0.02548	0.13325	0.6627
Paludibacterium	400060	0.11018	0.02532	0.16125	0.44072
Pedomicrobium	47494	0.08551	0.02515	0.12006	1.36818
Megasphaera	906	0.12113	0.02506	0.35874	108.28837
Thermocrinis	75905	0.02429	0.02429	0.00978	0.04857
Mogibacterium	86331	0.04099	0.02404	0.06867	37.05549
Phenylobacterium	20	0.3204	0.02394	0.85275	7.04877
Mucilaginibacter	423349	0.02909	0.02305	0.0256	0.14546

Tannerella	195950	0.08902	0.02304	0.22556	78.06714
Rhizorhapis	1649487	0.023	0.023	0.0108	0.04601
Thermobaculum	262406	0.16496	0.02271	0.25602	4.45393
Cupriavidus	106589	0.30267	0.02219	0.80326	15.13353
Persicobacter	59740	0.02208	0.02208	0.00578	0.04417
Janthinobacterium	29580	0.06363	0.0218	0.08524	1.27255
Spiribacter	1335745	0.02166	0.02166	0.02136	0.04331
Lautropia	47670	0.10815	0.02065	0.68018	82.94759
Dechloromonas	73029	0.1725	0.02063	0.31559	2.41504
Trichococcus	82802	0.02941	0.01968	0.03365	25.09085
Aquifex	2713	0.01911	0.01911	0.01223	0.03823
Mesoplasma	46239	0.03928	0.019	0.03503	0.1964
Prochlorococcus	1218	0.01894	0.01894	0.00264	0.03788
Lachnoclostridium	1506553	0.09771	0.01779	0.3678	85.49867
Sphaerotilus	34102	0.01708	0.01708	0.01681	0.03416
Burkholderia	32008	0.23418	0.01706	0.83888	62.76101
Catenulispora	414878	0.0183	0.01703	0.01372	0.07318
Marivirga	869806	0.01657	0.01657	0.01551	0.03314
Ramlibacter	174951	0.0391	0.01652	0.04948	0.15642
Steroidobacter	469322	0.05863	0.01651	0.09568	1.9349
Nitrosopumilus	338191	0.01536	0.01628	0.01319	0.06145
Peptococcus	2740	0.12412	0.01626	0.27498	93.71041
Porphyrobacter	1111	0.16362	0.01625	0.25246	3.10887
Phaeospirillum	85265	0.01611	0.01611	0.01524	0.03221
Cloacibacterium	501783	1.43296	0.01609	4.76264	73.08116
Telluria	34069	1.21957	0.01583	4.55508	20.73268
Emticicia	312278	0.05205	0.01575	0.06273	0.36434
Myceligenerans	253183	0.01477	0.01567	0.00744	0.1329
Effusibacillus	1502725	0.04126	0.01563	0.05273	0.82526
Ralstonia	48736	0.22634	0.01556	3.47453	121.31977
Gracilibacillus	74385	0.01551	0.01551	0.01504	0.03103
Paraliobacillus	200903	0.01547	0.01547	0.00149	0.03094
Paucibacter	318147	0.01453	0.01543	0.0051	0.04358
Enterobacter	547	0.02263	0.01536	0.02749	0.42993

Akkermansia	239934	0.33826	0.01529	0.73812	35.51756
Filifactor	44259	0.06336	0.01493	0.19414	40.23129
Uliginosibacterium	392735	0.01622	0.01477	0.01421	0.11353
Methylorubrum	2282523	0.20731	0.01473	0.45147	12.43884
Polynucleobacter	44013	0.24755	0.01465	0.6057	1.98041
Persephonella	182899	0.02561	0.0145	0.02982	0.38411
Cutibacterium	1912216	0.99317	0.0145	3.67808	161.88665
Mesoflavibacter	444051	0.0144	0.0144	0.00314	0.02881
Rhodoferax	28065	0.01523	0.01414	0.01275	0.21326
Chelatococcus	28209	0.05559	0.01406	0.0867	0.27796
Roseimaritima	1579506	0.01997	0.01398	0.02025	0.09983
Ochrobactrum	528	0.08651	0.01393	0.18926	11.85131
Rubinisphaera	1649490	0.053	0.01361	0.06185	0.15901
Ferroplasma	74968	0.01344	0.01344	0.01262	0.02689
Hydrogenophilus	70774	0.10471	0.01316	0.24323	1.88483
Maribacter	252356	0.05468	0.01303	0.06611	0.38276
Singulisphaera	466152	0.01703	0.01298	0.01538	0.15328
Gloeobacter	33071	0.1251	0.01292	0.31216	1.50115
Labrenzia	478070	0.01286	0.01286	0.00151	0.02573
Lysobacter	68	0.02692	0.01285	0.03928	0.34999
Pseudomonas	286	0.78908	0.01268	4.72885	453.71842
Thermophagus	1434049	0.01131	0.0124	0.00843	0.03392
Advenella	290425	0.01379	0.01232	0.01005	0.0965
Cnuella	1648511	0.62997	0.01207	0.88143	1.8899
Aquiflexum	280472	0.01183	0.01183	0.00975	0.02365
Rhodovibrio	85274	0.01179	0.01179	0.00978	0.02358
Kingella	32257	0.02896	0.01139	0.11106	24.58706
Chloroflexus	1107	0.01777	0.01129	0.01748	0.1066
Herbaspirillum	963	2.14059	0.01082	7.4105	667.86312
Microbacterium	33882	0.28103	0.01054	0.68308	23.32563
Gemmobacter	204456	0.01043	0.01043	0.00791	0.02086
Promicromonospora	43676	0.01293	0.01033	0.01197	0.05171
Staphylococcus	1279	0.17	0.01032	1.46461	152.65728
Meiothermus	65551	0.08228	0.0103	0.12838	0.6582

Cardiobacterium	2717	0.01941	0.01017	0.03054	14.40299
Parageobacillus	1906945	0.08416	0.01015	0.22356	1.17819
Rheinheimera	67575	0.11019	0.01002	0.3015	3.08523

Table S1. Full list of 168 genera detected in ≥ 2 out of 1054 lung-relevant samples with median relative abundances ≥ 0.01%. Accessed the website of "https://mbodymap.microbiome.cloud/#/mbodymap" on July 3, 2022.
